# TLR2 and TLR4 triggering exerts contrasting effects with regard to HIV-1 infection of human dendritic cells and subsequent virus transfer to CD4^+ ^T cells

**DOI:** 10.1186/1742-4690-6-42

**Published:** 2009-05-06

**Authors:** Sandra Thibault, Rémi Fromentin, Mélanie R Tardif, Michel J Tremblay

**Affiliations:** 1 Faculté de Médecine, Université Laval, Québec, Canada; 2 Centre de Recherche en Infectiologie, Centre Hospitalier de l'Université Laval, Québec, Canada

## Abstract

**Background:**

Recognition of microbial products through Toll-like receptors (TLRs) initiates inflammatory responses orchestrated by innate immune cells such as dendritic cells (DCs). As these cells are patrolling mucosal surfaces, a portal of entry for various pathogens including human immunodeficiency virus type-1 (HIV-1), we investigated the impact of TLR stimulation on productive HIV-1 infection of DCs and viral spreading to CD4^+ ^T cells.

**Results:**

We report here that engagement of TLR2 on DCs increases HIV-1 transmission toward CD4^+ ^T cells by primarily affecting *de novo *virus production by DCs. No noticeable and consistent effect was observed following engagement of TLR5, 7 and 9. Additional studies indicated that both HIV-1 infection of DCs and DC-mediated virus transmission to CD4^+ ^T cells were reduced upon TLR4 triggering due to secretion of type-I interferons.

**Conclusion:**

It can thus be proposed that exposure of DCs to TLR2-binding bacterial constituents derived, for example, from pathogens causing sexually transmissible infections, might influence the process of DC-mediated viral dissemination, a phenomenon that might contribute to a more rapid disease progression.

## Background

Myeloid dendritic cells (mDCs) play a dominant role in the induction and regulation of the adaptive immune response. It has been demonstrated that immature mDCs reside in submucosal tissues that are in contact with the external environment. These cells act as sentinels and continuously patrol the surrounding environment to detect potential invaders. Upon encountering a pathogen, they scavenge and internalize the intruder before migrating to the draining lymph nodes, where they present processed antigens to CD4^+ ^T cells, thus initiating an immune response [1].

Pathogens express signature motifs better known as pathogen-associated molecular patterns (PAMPs), which are recognized by immature mDCs through several pathogen-recognition receptors [2,3] such as Toll-like receptors (TLRs) [4,5]. These specialized receptors provide a first line of defence against a pathogen attack and rapidly activate defence signalling pathways following initial infection. TLRs are considered as playing a crucial role in the switch from innate to adaptive immunity in mammals. To date, at least 10 distinct TLRs have been characterized in humans and they are classified according to which PAMPs they recognize [6]. For example, TLR2, 4 and 5 mainly recognize bacterial components, whereas TLR3, 7, 8 and 9 detect nucleic acids derived from microorganisms [7]. The detection of PAMPs by TLRs triggers biochemical events resulting in NF-κB activation and induction of a pro-inflammatory response. The latter phenomenon is characterized by the migration of immature mDCs to secondary lymphoid organs where they mature and efficiently present the nominal antigen to CD4^+ ^T cells [1,8-10].

Due to their strategic localization in mucosal epithelia, immature mDCs are among the first cells to encounter HIV-1 after sexual transmission [11-14], and they are thought to play a crucial role during the initial stages of virus infection and dissemination [15]. HIV-1 can productively infect immature mDCs, although not at a rate sufficient to affect viral load. Nonetheless, this cell subpopulation contributes to viral propagation, as they migrate to lymph nodes, where they efficiently transfer newly produced virions to CD4^+ ^T cells through the immunological synapse [16]. This specific type of virus propagation is called transfer in *cis *or late transfer. Another type of transfer can take place when virions, either surface-bound or inside intracellular vesicles, are released following an intimate contact between DCs and CD4^+ ^T cells. This type of virus transmission is termed transfer in *trans *or early transfer [17,18]. Thus, by capturing HIV-1 at sites of viral entry into the body and transferring viruses to CD4^+ ^T cells, immature mDCs may be critical to the process of HIV-1 transmission.

The impact of microbial products on HIV-1 pathogenesis was highlighted by recent studies showing that acute HIV-1 infection increases the gut permeability favouring translocation of microbial products through the intestinal barrier into submucosal lamina propria and then mesenteric lymph nodes and bloodstream [19-23]. This phenomenon causes systemic immune activation that will in turn promote HIV-1 infection and spreading. In addition to HIV-1, several other factors can lead to enhanced microbial translocation across the intestinal barrier including direct injury of epithelial cells by others pathogens or toxins that increase the gut permeability. Translocation of microbial products can also increase activation of mDCs in the lamina propria through TLR stimulation. Some studies have previously monitored the impact of TLR stimulation on DCs. For example, activation of DCs by lipoproteins derived from *Porphyromonas gingivalis *and *Mycoplasma fermentans *was found to be mediated by TLR2 [24,25]. Moreover, stimulation of TLR4, 7 and 9 in DCs has been reported to lead to secretion of type-I interferons (IFNs) such as IFNα and IFNβ, two soluble factors that can repress HIV-1 replication. It has been demonstrated that type-I IFNs display pleiotropic effects which affect several steps in the virus life cycle from the initial viral uptake to the release of newly formed virions [26-29]. However, we are only beginning to study the putative effect(s) of bacterial products that can bind TLRs in DCs in the context of HIV-1 infection [30,31]. It has been recently reported that productive HIV-1 infection of immature monocyte-derived DCs is enhanced following TLR2 engagement by *Neisseria gonorrhoeae *[30].

Considering the key role played by mDCs in the pathogenesis of HIV-1 infection, that mDCs are constantly exposed to microbial components derived from different pathogens and commensal microorganisms upon microbial translocation, and knowing that this phenomenon accentuates HIV-1 infection and spreading, we investigated whether TLR2, 4, 5, 7 and 9 agonists can directly modulate the ability of immature monocyte-derived DCs (IM-MDDCs), which are considered as myeloid-like DCs, to be productively infected with HIV-1 and transfer virus to susceptible CD4^+ ^T cells.

## Results

In this study, we made use of agonists specific for various TLRs known to be expressed in DCs, namely Pam3Csk4 and LTA for TLR2, LPS for TLR4, flagellin for TLR5, R837 for TLR7, and bacteria-derived unmethylated DNA for TLR9. Our experiments were all performed with immature DCs because these cells have been proposed to be among the first potential targets that encounter HIV-1 during sexual transmission and also because virus replication is very inefficient in mature DCs. Importantly, IM-MDDCs were selected based on the observation that their characteristics resemble those of the different DC subsets found *in vivo *(e.g. mDCs, immature dermal DCs and interstitial DCs) [32-34], including their TLR expression patterns [35].

### TLR2 triggering affects primarily de novo virus production in IM-MDDCs

**Figure 1:**
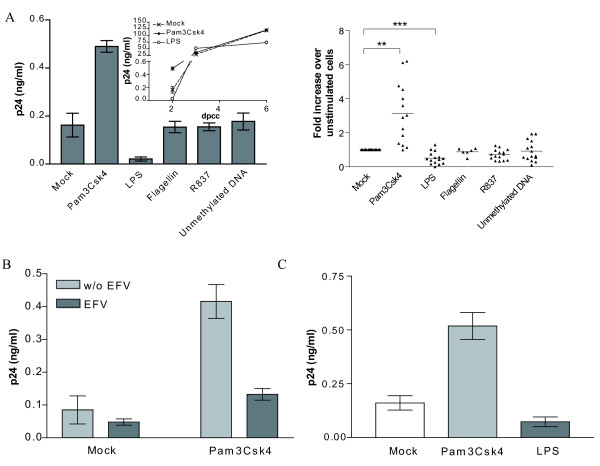
**TLR2 and 4 triggering modulates HIV-1 transfer between IM-MDDCs and CD4^+ ^T cells**. A) IM-MDDCs were either left untreated (mock) or stimulated for 2 hours with the following TLR agonists: Pam3Csk4 (5 μg/ml), LPS (0.1 μg/ml), flagellin (5 μg/ml), R837 (5 μg/ml) and unmethylated DNA (5 μg/ml). Cells were then pulsed with NL4-3/Bal*env *and co-cultured with autologous CD4^+ ^T cells. Finally cell-free supernatants were harvested at 2, 3 and 6 days post-coculture (dpcc) and the viral content was assessed by a p24 assay. Data depicted in the left panel represent the mean ± standard deviations of quadruplicate samples from a representative single donor at 2 dpcc, whereas the kinetics of virus production for the same donor are displayed in the small insert. Results from multiple different donors are illustrated in the right panel (2 dpcc) (**: P < 0.01; ***: P < 0.001). B) IM-MDDCs were initially either left untreated or treated with EFV. Thereafter, cells were either left untreated or treated with Pam3Csk4. Data shown represent the mean ± standard deviations of quadruplicate samples from a single donor at 2 dpcc and are representative of 8 distinct donors. C) A similar experimental approach was used except that transfer studies were carried out with the X4-tropic strain NL4-3. Data shown represent the mean ± standard deviations of quadruplicate samples from a single donor at 2 dpcc and are representative of 3 different donors.

To define whether TLR stimulation can affect HIV-1 transfer, IM-MDDCs were first treated for only 2 hours with one of the tested TLR agonists before pulsing with the R5-using HIV-1 strain NL4-3/Bal*env*. Thereafter, the cell-virus mixture was co-cultured with autologous CD4^+ ^T cells and cell-free supernatants were harvested at 2, 3 and 6 days post-co-culture (dpcc) to measure virus transfer. As depicted in Fig. 1A (left panel), transmission of HIV-1 was markedly increased upon TLR2 stimulation at 2 dpcc, whereas a diminution was seen following LPS-mediated engagement of TLR4. The kinetics of virus production in the co-cultures revealed that TLR2 and 4 triggering affects an early step(s) in the process of virus transfer since the modulatory effects were disappearing over time (small insert in the left panel). Engagement of TLR5, 7 and 9 did not affect the DC-mediated propagation of HIV-1. Similar patterns of HIV-1 transfer were obtained when experiments were conducted with multiple independent donors (Fig. 1A, right panel). Next, we evaluated whether the observed modulation of virus transfer could be attributable to *de novo *virus production by IM-MDDCS (i.e. late transfer). This issue was solved by adding the inhibitor of reverse transcription Efavirenz (EFV) before pulsing IM-MDDCs with virions. Results illustrated in Fig. 1B indicate that the TLR2-mediated signal transduction pathway was affecting primarily direct productive infection of IM-MDDCs (i.e. late transfer due to newly formed viral entities) since the Pam3Csk4-dependent augmentation in virus transfer was almost totally abrogated upon treatment with EFV. Although it is well accepted that primary HIV-1 infection is caused by R5-tropic viruses, some experiments were also carried out with an X4-using isolate of HIV-1 (i.e. NL4-3). The TLR2-mediated enhancement in virus transfer was also seen with the X4-tropic variant as well as the reduction of HIV-1 propagation by the TLR4 agonist (Fig. 1C). The effect of the studied TLR agonists on cell viability was also monitored using the fluorescent cytotoxic MTS assay. Cell viability was not affected by the studied TLR ligands used at concentrations known to modulate the DC-mediated transfer of HIV-1 (data not shown).

**Figure 2:**
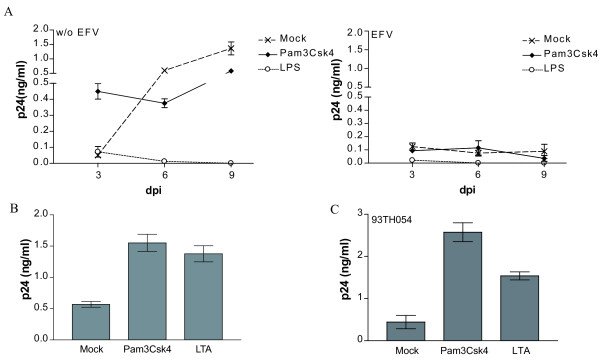
**De novo virus production in IM-MDDCs is affected by TLR2 and 4 engagement**. (A) IM-MDDCs were either left untreated (mock) or stimulated for 2 hours with the listed TLR agonists. Thereafter, cells were washed twice and pulsed with NL4-3/Bal*env*. IM-MDDCs were either left untreated (left panel) or treated with EFV (right panel) before addition of TLR agonists. Supernatants were harvested at 3, 6 and 9 days post-infection (dpi) and the viral content was monitored by a p24 test. Data depicted represent the mean ± standard deviations of quadruplicate samples from a single donor and are representative of 3 different donors. (B) A similar experimental strategy was used except that cells were either left untreated or exposed to the listed TLR2 ligands. Data shown represent the mean ± standard deviations of quadruplicate samples from two different donors (3 dpi). (C) Cells were either left untreated (mock) or stimulated for 2 hours with the listed TLR2 agonists. Thereafter, cells were washed twice and pulsed with the clinical HIV-1 isolate 93TH054. Supernatants were harvested at 5 dpi and the viral content evaluated by a p24 test. The data shown represent the mean of quadruplicate samples from 2 different donors.

To corroborate the role played by TLR2/4 triggering in late virus transfer, we measured the effect of TLR2 and 4 ligands upon acute virus infection of IM-MDDCs. As expected, virus production in IM-MDDCs cultured alone was much lower than in co-cultured cells (Fig. 2A, left panel). Interestingly, replication of HIV-1 in IM-MDDCs was still enhanced by the TLR2 ligand at an early time point following virus infection while engagement of TLR4 led to a potent inhibition of virus production. Again, flagellin (TLR5), R837 (TLR7) and unmethylated DNA (TLR9) showed no noticeable and consistent effect on HIV-1 replication in IM-MDDCs cultured alone (data not shown). The TLR2-mediated increase in *de novo *virus production in IM-MDDCs was no longer seen in presence of EFV (Fig. 2A, right panel), thus confirming that the effect was primarily due to *cis *replication in the DC population. To provide additional *in vivo *significance to our findings and considering that Pam3Csk4 is a synthetic TLR2 agonist, we also tested the effect of the prototypic TLR2 agonist LTA that was isolated directly from *Staphylococcus aureus*. Results depicted in Fig. 2B illustrate that both TLR2 agonists, i.e. synthetic and isolated bacterial constituent, can increase virus production in IM-MDDCs cultured alone. To more closely parallel natural conditions, acute infection experiments were also conducted with a R5-tropic field isolate of HIV-1 (i.e. 93TH054). As shown in Fig. 2C, both Pam3Csk4 and LTA were able to enhance replication of the clinical isolate 93TH054 in IM-MDDCs.

### TLR2, 4 and 5 triggering results in nuclear translocation of NF-κB

**Figure 3:**
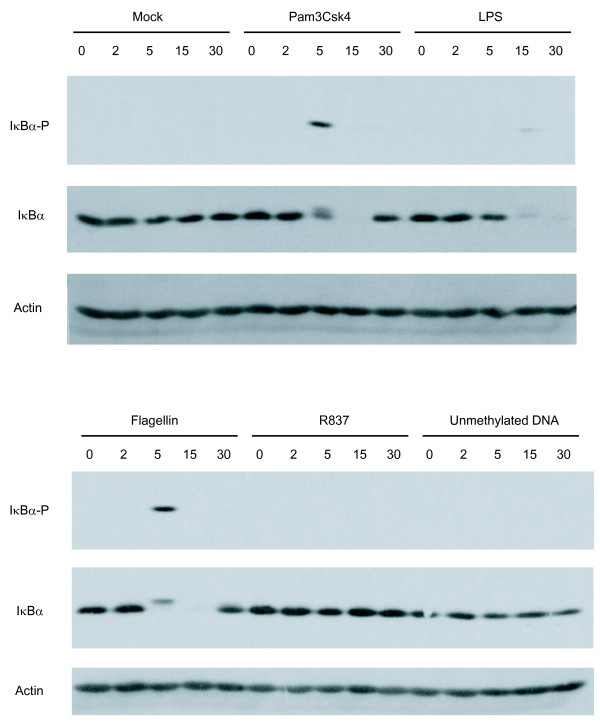
**NF-κB is activated in IM-MDDCs following TLR2, 4 and 5 triggering**. Cells were either left untreated (mock) or stimulated for 0, 2, 5, 15 and 30 min with the listed TLR ligands. Cells were then lysed and proteins were loaded on a 12% SDS-polyacrylamide gel, transferred to a membrane, and revealed by anti-phospho-IκBα, anti-IκBα, or anti-actin. Data from a single donor representative of 4 different donors are displayed.

The transcription factor NF-κB is recognized as a powerful inducer of HIV-1 transcription and gene expression due to the presence of two NF-κB binding sites located within the enhancer domain. Therefore, we next studied the possible TLR2-, 4-, 5-, 7- and 9-mediated induction of NF-κB by analyzing the phosphorylation state of IκBα, a sign of NF-κB activation. IM-MDDCs were stimulated with the studied TLR agonists for 0, 2, 5, 15 and 30 minutes and lysed. Phosphorylation and degradation of IκBα were monitored by western blotting analyses. Data shown in Fig. 3 demonstrate that IκBα is rapidly phosphorylated following TLR2, 4 and 5 triggering. For example, a band specific for the phosphorylated form of IκBα was detected following 5 minutes of exposure of IM-MDDCs to the TLR2 agonist. This rapid IκBα phosphorylation was accompanied by a fast and extensive degradation of IκBα at 5 and 15 minutes. A weaker but detectable phosphorylation of IκBα was also seen upon engagement of TLR4, but this time, 15 minutes following treatment with the agonist. The degradation of IκBα was also delayed, as compared to TLR2 triggering, since the protein started to disappear only 15 minutes after treatment. Furthermore, engagement of TLR5 resulted in a pattern of IκBα phosphorylation and degradation comparable to the situation prevailing in the presence of TLR2 ligand. TLR7 and 9 triggering resulted in little impact on IκBα, which is not surprising considering the reported low expression levels of TLR7 and 9 in IM-MDDCs [35,36].

### Soluble factors are released upon engagement of the tested TLRs in IM-MDDCs

**Figure 4:**
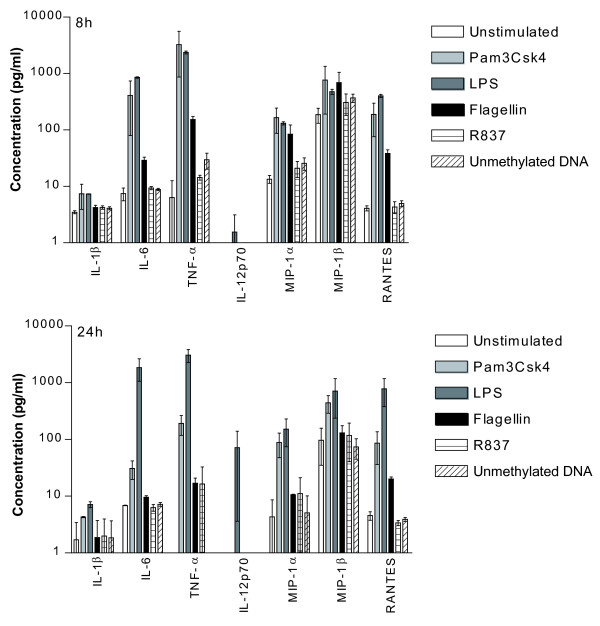
**Some cytokines and chemokines are secreted following engagement of TLRs**. IM-MDDCs were either left untreated or stimulated for 8 and 24 hours with the listed TLR ligands. Cell-free supernatants were harvested and analyzed with a Bio-Plex assay that can detect all the indicated soluble factors. The results shown are representative of two separate experiments performed with two distinct donors.

Upon exposure to some microbial products, DCs can produce pro-inflammatory cytokines and chemokines that influence the nature of the immune response. The functionality of the studied TLRs was assessed by measuring the production of some defined soluble factors. As illustrated in Fig. 4, TLR2, 4 and 5 ligands induce significant secretion of IL-6, TNF-α, MIP-1α and RANTES. The IL-12p70, which is the bioactive form of IL-12 involved in a T_H_1 response, has only been detected in supernatants harvested from LPS-stimulated IM-MDDCs. A weak production of TNF-α, MIP-1α and MIP-1β was also seen when using TLR7 and 9 agonists.

### TLR2 and 4 triggering modulates an early step in HIV-1 replication

**Figure 5:**
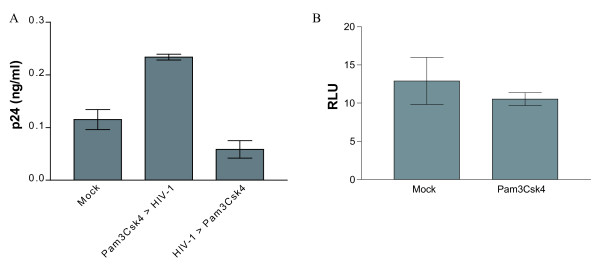
**TLR2 stimulation influences an early step in HIV-1 life cycle**. A)IM-MDDCs were either left untreated (mock) or stimulated for 2 hours with the TLR2 agonist Pam3Csk4 (5 μg/ml) before or after exposure for 1 hour to NL4-3/Bal*env*. Supernatants were harvested at 72 hours post-infection and the viral content was evaluated by a p24 test. B) Cells were infected with VSV-G pseudotyped reporter viruses for 2 hours, washed twice and put in culture for 48 hours. Next, IM-MDDCs were either left untreated (mock) or stimulated for 2 hours with Pam3Csk4. Cells were then washed twice, cultured for 48 hours and lysed to monitor luciferase activity (expressed in relative light units/RLU). The data shown represent the mean ± standard deviations of quadruplicate samples from a single donor and are representative of 3 distinct donors.

To provide information on the mechanism(s) by which TLR2 engagement can promote virus production, IM-MDDCs were either treated first with the TLR2 agonist prior to virus infection or, alternatively, pulsed first with HIV-1 before Pam3Csk4 treatment. As shown in Fig. 5A, a TLR2-mediated enhancement of virus replication was seen only when stimulation took place before HIV-1 infection, thus suggesting that the signalling cascade triggered by the agonist acts most likely at an early step in the virus life cycle. To confirm that TLR2 triggering is not affecting more downstream events in HIV-1 replication (i.e. subsequent to integration), IM-MDDCs were infected with single-cycle reporter virus pseudotyped with VSV-G for a time period sufficient to allow integration of the viral genetic material within host genome (i.e. 48 hours) [37]. The use of such viruses prevents re-infection events and bypasses the natural mode of HIV-1 entry (namely via a CD4- and CCR5-dependent pathway) [38]. Thereafter, cells were treated with Pam3Csk4 before monitoring the virus-directed luciferase activity. Results from Fig. 5B demonstrated that integrated viral DNA was not activated upon engagement of TLR2, thus corroborating that TLR2 triggering is primarily affecting an early event in the virus life cycle (i.e. before virus integration).

**Figure 6:**
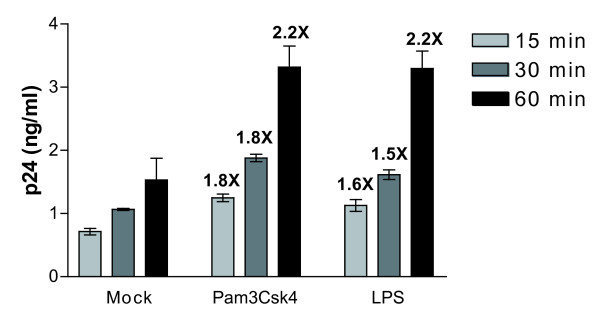
**TLR2 and 4 triggering increases viral entry in IM-MDDCs**. A) IM-MDDCs were either left untreated (mock) or treated with TLR2 and 4 ligands for 2 hours and washed twice. Then, cells were pulsed with NL4-3/Bal*env *for 15, 30 and 60 min at 37°C. Next, the virus-cell mixture was washed extensively with PBS and trypsinized to remove uninternalized virus. Finally, cells were lysed and the p24 contents were measured by ELISA. Numbers depicted above bars represent fold increase relative to p24 levels in untreated control cells (considered as 1). The data shown represent the mean ± standard deviations of triplicate samples from 3 different donors.

**Figure 7:**
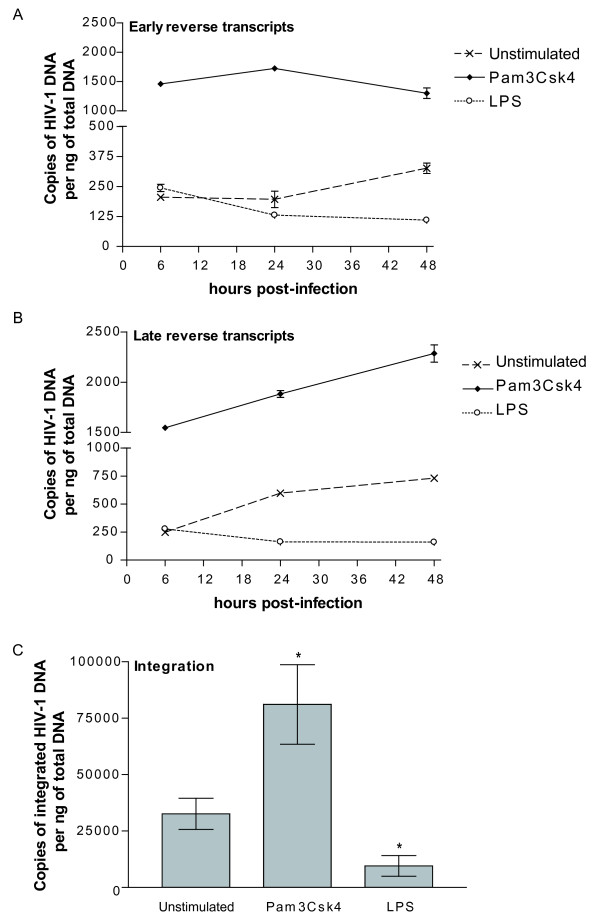
**Early steps in HIV-1 replication are modulated by TLR2 and 4 agonists**. IM-MDDCs were either left untreated or stimulated for 2 hours with the TLR2 or 4 agonist. Cells were next pulsed with NL4-3/Bal*env *for 1 hour, washed twice and incubated at 37°C. Total DNA was extracted at 6, 24 or 48 hours post-infection and used for the detection and quantification of early reverse transcripts (A), late reverse transcripts (B) and integrated viral DNA (C) using a real-time PCR method. The number of HIV-1 copies was determined by a standard curve prepared with the NL4-3/Bal*env *vector. Data depicted in panels (A) and (B) represent the mean ± standard deviations of duplicate samples representative of two distinct donors whereas those illustrated in panel (C) represent the mean ± standard deviations of duplicate samples from 5 (Pam3Csk4) or 3 separate donors (LPS) (*: P < 0.05).

To shed light on the mechanism(s) by which TLR2 and 4 triggering can affect *de novo *virus production, the extent of virus entry was quantified in IM-MDDCs. Data displayed in Fig. 6 indicate that virus internalization was increased at a comparable level by TLR2 and 4 agonists as compared to untreated cells. Since there is no linear relationship between internalization of viral particles in IM-MDDCs and productive infection, we investigated whether reverse transcription and integration processes are affected by a treatment with Pam3Csk4 and LPS. This issue was addressed through the use of a quantitative real-time PCR assay that has been described previously by Zack and colleagues [39]. Results displayed in Fig. 7A indicate that the amounts of early reverse transcripts were increased by a treatment with the TLR2 agonist while a diminution was seen with LPS. A similar trend was made when measuring the levels of late reverse transcripts (Fig. 7B). Integration of viral DNA was also promoted by the TLR2 agonist (Fig. 7C), whereas this process was significantly reduced upon a treatment with the TLR4 ligand LPS.

### TLR4 stimulation induces secretion of type-I IFNs

**Figure 8:**
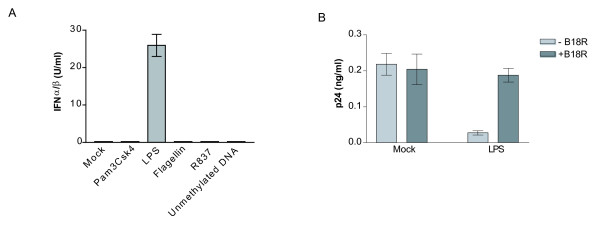
**TLR4-mediated decrease in de novo virus production involves type-I IFNs**. A) IM-MDDCs were either left untreated (mock) or stimulated for 6 hours with the listed TLR ligands. Cell-free supernatants were harvested and the levels of IFNα/β were quantified through the use of HEK-Blue™ IFNα/β cells. The data shown represent the mean ± standard deviations of quadruplicate samples from a single donor. B) IM-MDDCs were either left untreated (mock) or stimulated for 2 hours with the TLR4 ligand LPS. Cells were then washed twice, pulsed with NL4-3/Bal*env *and cultured in absence or presence of B18R (0.1 μg/ml). Supernatants were harvested at 72 hours post-infection and the viral content was evaluated by a p24 test. The data shown represent the mean ± standard deviations of quadruplicate samples from a single donor and are representative of 4 different donors.

Knowing that TLR4 stimulation can lead to secretion of type-I IFNs (i.e. IFNα and IFNβ), we next wanted to see whether the observed TLR4-dependent diminution in virus replication was attributable to these antiviral agents. To demonstrate the participation of type-I IFNs in the LPS-dependent modulatory effect on virus production in IM-MDDCs, we performed experiments with HEK-Blue™ IFNα/β indicator cells. Results depicted in Fig. 8A indicate that the TLR4 ligand LPS acted as a strong inducer of IFNα/β in IM-MDDCs while TLR2, 5, 7 and 9 triggering did not result in the secretion of type-I IFNs. To confirm the involvement of the LPS-mediated production of type-I IFNs in the observed diminution of HIV-1 production in IM-MDDCs, we performed studies with B18R, a vaccinia virus-derived soluble receptor that blocks the effect of biologically functional type-I IFNs (e.g. IFNα, IFNβ and IFNω). Results depicted in Fig. 8B indicate that the TLR4-mediated reduction in *de novo *virus production seen in IM-MDDCs was indeed associated with secretion of type-I IFNs.

## Discussion

It is now well established that the majority of HIV-1 infections are acquired sexually. Considering that co-infections exacerbate the risk for HIV-1 acquisition, it is relevant to understand how microbial constituents can modulate the process of virus infection and create a more favourable environment for HIV-1 dissemination. Immature mDCs residing in mucosal tissues are thought to be one of the first cell types encountering HIV-1 during sexual transmission. These cells can efficiently capture HIV-1 and depending on the receptors used and the surrounding environment, several distinct processes can occur concurrently. For example, viruses can directly bind CD4 and the appropriate co-receptor on the plasma membrane of immature mDCs leading to a cytosolic delivery of viral material and productive infection [40,41]. Alternatively, incoming virions can either remain in an infectious state within intracellular vesicles or be associated in membrane protrusions and microvilli found on the plasma membrane before a subsequent transmission through the virological synapse [17,42,43]. Internalized viruses can also be degraded by lysosomal enzymes inside the endosomal machinery [44]. It is thus expected that exposure of immature mDCs to stimuli such as microbial-derived PAMPs might influence the virus uptake pathway and the eventual fate of HIV-1 in these cells.

In the present study, we investigated whether TLR2, 4, 5, 7 and 9 triggering can modulate the ability of IM-MDDCs to capture, internalize, replicate and transfer HIV-1. We first assessed the capacity of the tested agonists to modulate HIV-1 transmission. We found that the TLR2 ligand Pam3Csk4 increased virus transfer from IM-MDDCs to autologous CD4^+ ^T cells, whereas the process remains almost unaffected upon TLR5, 7 and 9 triggering. Moreover, we report that the process of HIV-1 propagation in a co-culture system was diminished by TLR4 engagement. The fact that the TLR2- and 4-mediated effect on HIV-1 propagation was seen only at an early time point following initiation of the co-culture (i.e. 2 days) and was rapidly lost thereafter is indicative of a modulatory effect on intricate interactions between HIV-1 and IM-MDDCs. The loss of the TLR2- and 4-dependent effect on HIV-1 transfer at later time points following initiation of the culture is due to a rapid and massive spreading of HIV-1 in the CD4^+ ^T cell population. The validity and clinical relevance of our findings are provided by three sets of experiments. First, the TLR2-mediated augmentation in virus production was detected when using both a synthetic (i.e. Pam3Csk4) and a more natural TLR2 agonist (i.e. LTA). Second, similar findings were made when viral infection studies were carried out with a field isolate of HIV-1. Third, the TLR2-dependent up-regulatory effect on HIV-1 propagation was seen with both R5- and X4-using virions.

To define the exact contribution of *de novo *virus production from IM-MDDCs in the TLR2-dependent up-regulatory effect on HIV-1 transfer, co-culture experiments were performed in presence of EFV. According to our results, it can be proposed that TLR2 triggering is mostly affecting the direct productive infection of IM-MDDCs with HIV-1 since treatment with EFV reduced the level of viral transfer close to that observed for untreated cells. Acute infection studies performed with IM-MDDCs confirmed that TLR2 engagement is modulating primarily *de novo *virus production in this cell type. Our findings are perfectly in line with a recent study showing that the TLR2 ligand Pam3Csk4 strongly enhanced HIV-1 transmission [45]. However, the reported TLR2-mediated enhancement in virus transmission was due to a more important HIV-1 capture and not to a superior virus replication in this cell type as we demonstrate here in the present work. Differences in experimental methodologies may account for the discrepant results. Indeed, the experimental cell system used by de Jong and colleagues consisted of human epidermal sheet explants that contained resident Langerhans cells. It should be noted that Langerhans cells and IM-MDDCs are quite distinct with respect to their cell surface expression patterns, migratory capacity, endocytic ability and immunological functions [46]. Our results are also consistent with findings published by Zhang and colleagues who have demonstrated that HIV-1 replication in IM-MDDCs is promoted by *Neisseria gonorrhea *mainly through engagement of TLR2 by the peptidoglycan of the gonococci [30]. However, in this study, IM-MDDCs were first exposed for 48 hours to bacteria or other *Neisseria gonorrhea *constituents before HIV-1 pulsing as opposed to 2 hours in the present study. It is expected that the DC population used by Zhang and co-workers is displaying a more mature phenotype than what we have used in our experiments.

Sensing PAMPs through TLRs usually triggers signalling cascades resulting in the activation of the transcription factor NF-κB and the induction of pro-inflammatory responses, which are required to fight the invaders. It is well known that induction of NF-κB drives HIV-1 transcription and production of newly synthesized virions in both CD4^+ ^T lymphocytes and monocytes/macrophages (reviewed in [47,48]). Although the exact role played by NF-κB in the process of acute HIV-1 infection of IM-MDDCs remains unclear, we hypothesized that it is the same for all myeloid lineage cells. In order to define if the tested TLR agonists can trigger signalling cascades resulting in NF-κB activation, we measured phosphorylation and ensuing degradation of the natural repressor of NF-κB (i.e. IκBα). Our results indicate that IκBα is rapidly phosphorylated following TLR2 stimulation. Although DC-mediated virus transfer was not modulated upon TLR5 triggering, a potent induction of NF-κB was seen following ligation of TLR5. It can thus be proposed that there is no direct relationship between these two events. This postulate is confirmed by our findings that TLR4 signalling results in a quite different outcome since a reduced HIV-1 propagation was detected concomitantly with an induction of NF-κB. Moreover, our observations that the NF-κB-regulated cytokine TNF-α is induced, albeit at different levels, by all studied TLR ligands supports this hypothesis. Nevertheless, based on results obtained with a Bio-Plex assay, it is obvious that TLR2 and 4 triggering in IM-MDDCs is more efficient than TLR5, 7 and 9 stimulations as evidenced by the higher production of IL-6, TNF-α and RANTES.

Knowing that TLR4 stimulation can activate pathways resulting in both NF-κB activation and secretion of type-I IFNs [49,50], we hypothesized that the observed TLR4-mediated inhibition of virus production in IM-MDDCs is linked to the production of such soluble factors. It has already been reported that exposure of macrophages to LPS or gonococcal lipooligosaccharide reduces HIV-1 replication through a mechanism relying on production of type-I IFNs [51-53]. We showed here the direct involvement of type-I IFNs in TLR4-dependent decrease in HIV-1 replication through the use of the recombinant B18R protein and HEK-Blue™ IFNα/β cells. Interestingly, data from HEK-Blue™ IFNα/β cells indicate that LPS treatment leads to a rapid production of type-I IFNs (i.e. as early as 2 hours following exposure to the TLR4 ligand) (data not shown) reaching a peak at 6 hours. Given that IM-MDDCs were inoculated with HIV-1 at 2 hours after addition of LPS, it can be proposed that the initial steps in the virus life cycle are affected by IFNα and/or β. Data from studies performed with B18R suggest that secretion of type-I IFNs, which is seen following TLR4 triggering, may counteract the likely positive effect of NF-κB on virus gene expression. Surprisingly, the process of virus entry was enhanced upon LPS treatment. It is likely that the positive impact of TLR4 ligand in HIV-1 entry is totally neutralized by the antiviral activity of IFNα/β.

The LPS-mediated diminution in HIV-1 transmission contrasts with some previous studies reporting that the DC-mediated virus transfer is enhanced upon LPS treatment [16,54-61]. However, in these studies, IM-MDDCs were exposed to LPS for at least 24 to 48 hours before HIV-1 pulsing and the initiation of the co-culture with CD4^+ ^T cells. This time period is sufficient to induce a complete maturation phenotype in DCs. In our study, we treated IM-MDDCs with LPS for only 2 hours, which is not sufficient *per se *to induce DC maturation. The present work was aimed at measuring the impact of TLR-mediated stimulation that is not long enough to obtain DC maturation but sufficient to trigger some biological responses such as cytoskeleton remodelling and an increase in macropinocytosis [62]. Interestingly, the shape of LPS-stimulated DCs is completely different at 2 and 24 hours following stimulation. Indeed, DCs acquire an elongated form and stick at the bottom of the well after a 2 hours treatment period whereas they form cellular aggregates remaining in suspension after 24 hours of treatment with LPS (unpublished data). Therefore, it is not surprising to obtain different results with the two conditions in regard to DC-mediated *trans*-infection of CD4^+ ^T cells with HIV-1. This is confirmed by previous findings since the efficiency of HIV-1 transmission is enhanced following maturation of DCs [8,16,54,63].

Considering the up-regulatory effect of NF-κB with regard to HIV-1 transcription and the potent induction of this transactivator by TLR2 stimulation, we thought that the TLR2-mediated augmentation in *de novo *virus production by IM-MDDCs would be similar if TLR2 triggering would occur after viral uptake. Surprisingly, virus production was not affected under such experimental conditions. This suggests that engagement of TLR2 in IM-MDDCs carrying integrated viral DNA is not sufficient *per se *to drive HIV-1 gene expression. Therefore, the signal transduction pathway that is engaged following TLR2 occupancy is affecting an early event in the replicative cycle of HIV-1 (i.e. prior to reverse transcription or integration). In an attempt to shed light on the exact mechanism(s) by which TLR2 triggering can increase HIV-1 productive infection of IM-MDDCs, we performed viral entry assays. We found that internalization of HIV-1 within IM-MDDCs was augmented upon treatment with both TLR2 and 4 agonists. This observation was unexpected in light of the TLR2-mediated secretion of CCR5-binding chemokines. However, it is possible MIP-1α, MIP-1β and RANTES could be released later than 2 hours following treatment with Pam3Csk4.

With regard to the TLR2-mediated enhancement in virus entry, several hypotheses can be proposed. Previous studies have revealed that HIV-1 entry into DCs can result either in cytosolic delivery that leads to productive infection [40,41], preservation into intracellular vesicles in an infectious state for a subsequent transmission through the virological synapse [17,42,43], or degradation by lysosomal enzymes in the endosomal compartments [44]. It can be hypothesized that the route of virus entry is affected upon TLR2 triggering. Knowing that the vast majority of viruses entering DCs is degraded rapidly (i.e. up to 95%) [44,56,64-66], it is possible that TLR2 stimulation increases the overall percentage of virions that can evade the degradation process by a yet to be defined mechanism. Alternatively, it can also be postulated that TLR2 triggering prior to virus exposure favors HIV-1 entry through a pH-independent fusion of viral and cellular membranes. It is known that this mechanism of HIV-1 internalization into target cells results in productive infection [40,41,67]. Interestingly, a previous study has shown that TLR2 engagement by a bacterial product results in recruitment of this pattern-recognition receptor within specialized microdomains called lipid rafts [68]. The lateral diffusion of TLR2 inside lipid rafts might result in a more efficient virus entry through such specific microdomains, which are recognized as a significant portal of entry for a broad range of pathogens including HIV-1 [69-73]. The possibility that TLR2 triggering is positively affecting the early steps of HIV-1 infection in IM-MDDCs is confirmed by quantitative measurements of reverse transcripts and integrated viral DNA copies. The negative impact of LPS on the most proximal events in HIV-1 replicative cycle has been confirmed by the quantitative real-time PCR test. These results are expected since type-I IFNs have been shown to exert a paracrine effect on virus infection by promoting RNA degradation and also by increasing the level of the restriction factor APOBEC3G [74-78]. Altogether our data suggest that a brief treatment of IM-MDDCs with LPS (i.e. 2 hours) is sufficient to induce the release of type-I IFNs that will prevent productive HIV-1 infection of this cell population.

We have also performed flow cytometry analyses to monitor CD4 and CCR5 expression following a treatment for 2 hours with TLR2 and 4 agonists but not in response to TLR5, 7 and 9 ligands based on the absence of a significant and reproducible effect of those agonists on HIV-1 infection and viral transmission. Our observations indicate that engagement of TLR2 and 4 down-regulates expression of both CD4 and CCR5 (data not shown). These results are not surprising given that one of the earliest responses to TLR agonists is an increase in membrane turnover and macropinocytosis [62]. However the TLR2- and 4-mediated reduced expression of CD4 and CCR5 cannot explain the opposite effects of these two ligands with regard to HIV-1 replication in IM-MDDCs cultured alone. Although it is clear that expression levels of HIV-1 receptor and coreceptors on the surface of DCs can affect virus entry, other factors can also modulate the life cycle of HIV-1 in DCs (e.g. distribution of viral receptor/coreceptors in some specific membrane microdomains, virus entry at the cell membrane or via endosomes, efficiency of reverse transcription and integration processes, modulation of restriction factors, etc.). Additional experiments are needed to solve this issue.

Under physiological conditions, immature mDCs are localized in mucosa-like genital and intestinal tracts and act as sentinels to prevent host invasion by certain pathogens. Upon a physical contact with an invader carrying PAMPs, immature mDCs become activated and migrate to the most proximal lymph nodes to prime CD4^+ ^T cells. The recognition of PAMPs by TLRs triggers intracellular signalling pathways, which culminate in secretion of proinflammatory cytokines, chemokines and type-I IFNs and maturation of DCs [79]. The genital mucosa is often in contact with external pathogens like *Neisseria gonorrhoeae, Chlamydia trachomatis *and *Treponema pallidum*, which respectively cause gonorrhea, chlamydial infection and syphilis. Those infections can damage the epithelial barrier and cause microbial translocation leading ultimately to inflammation and activation of mDCs and macrophages. In steady-state, resident flora of the vaginal mucosa is constituted primarily of lactobacilli that contribute to the equilibrium of the vagina flora by inhibiting harmful bacteria. However, when this equilibrium is broken (often following a pH decrease), the amount of lactobacilli is reduced and pathogenic bacteria will prevail. This phenomenon is common and results in a pathological condition called bacterial vaginosis (BV) [80]. This type of vaginosis is the most widespread, and about 50% of women are susceptible to this particular type of infection. It should be noted that BV is associated with an increased risk for contracting HIV-1 infection and several other sexually transmitted infections, including herpes simplex virus type 2 [81-83]. Moreover, BV is associated with increased levels of proinflammatory cytokines (e.g. IL-1β and IL-8) and these cytokines induce the secretion of other proinflammatory cytokines or recruit other immune cells, thus possibly increasing the number of cells permissive for HIV-1 infection [84,85]. Knowing this, it can be hypothesized that such bacteria-derived TLR ligands as well as others pathogen-encoded TLR agonists can modulate HIV-1 propagation by mDCs.

## Conclusion

In summary, this work provides new insights into the complex interconnections between HIV-1 and DCs. Our results reveal that some members of the TLR family (i.e. TLR2 and 4) can modulate the multifaceted interactions between HIV-1 and DCs.

## Methods

### Antibodies and reagents

Anti-phospho-IκBα and anti-IκBα were purchased from Cell Signaling (Beverly, Massachussets, USA), whereas anti-actin was obtained from Santa Cruz Biotechnology Inc. (Santa Cruz, California, USA). Hybridomas producing 183-H12-5C and 31-90-25, two antibodies recognizing different epitopes of the HIV-1 major viral core protein p24, were supplied by the AIDS Repository Reagent Program (Germantown, Maryland, USA) and ATCC (Manassas, Virginia, USA), respectively. Antibodies obtained from these cells were purified using mAbTrap protein G affinity columns according to the manufacturer's instructions (Amersham Pharmacia Biotech, Piscataway, New Jersey, USA). Pam3Csk4 (a synthetic tripalmitoylated lipopeptide that mimics the acylated amino terminus of bacterial lipoproteins) (TLR2 agonist), lipoteichoic acid (LTA) from *Staphylococcus aureus *(a purified bacterial component) (TLR2 agonist), ultra-purified lipopolysaccharide (LPS) (a purified bacterial component) (TLR4 agonist), flagellin (a purified bacterial component) (TLR5 agonist), R837 (an imidazoquinoline compound that mimics single-stranded RNA) (TLR7 agonist) and purified *E. coli DNA *(a purified bacterial component) (TLR9 agonist) were all purchased from InvivoGen (San Diego, California, USA). The anti-HIV-1 compound EFV was obtained through the AIDS Repository Reagent Program. IL-4 was purchased from R&D Systems (Minneapolis, Minnesota, USA) and GM-CSF was a kind gift from Cangene (Winnipeg, Massachussets, USA). The soluble vaccinia virus-encoded recombinant protein B18R was purchased from eBioscience (San Diego, CA). Phytohemagglutinin-L (PHA-L) and recombinant human IL-2 (rhIL-2) were obtained from Sigma (St-Louis, Missouri, USA) and AIDS Repository Reagent Program, respectively.

### Cells

Human embryonic kidney 293T cells were cultured in Dulbecco's modified Eagle's medium (DMEM) supplemented with 10% foetal bovine serum (FBS) (Wisent, St-Bruno, QC). HEK-Blue™ IFNα/β cells were purchased from InvivoGen (San Diego, CA) and cultured in DMEM supplemented with 10% FBS, zeocin (100 μg/ml) and blasticidin (10 μg/ml). Autologous CD4^+ ^T cells were isolated using a negative selection kit according to the manufacturer's instructions (Stem Cell Technologies Inc., Vancouver, BC). Purified CD4^+ ^T cells were cultured for five days in RPMI-1640 medium supplemented with 10% FBS before their activation with PHA-L (1 μg/ml) and rhIL-2 (30 U/ml) for two days. Monocytes (CD14^+^) were purified from freshly isolated peripheral blood mononuclear cells by immunomagnetic positive selection using the MACS CD14 micro beads kit (Stem Cell Technologies Inc). Purified CD14^+ ^cells were cultured in RPMI-1640 medium supplemented with 10% FBS, GM-CSF (1000 U/ml) and IL-4 (200 U/ml) for 7 days to obtain IM-MDDCs as previously described [86].

### Plasmids and production of viral stocks

pNL4-3 [87] and pNL4-3/Bal*env *[88] are full-length infectious molecular clones of HIV-1. In pNL4-3Bal*env*, the *env *gene of the X4 (T)-tropic NL4-3 strain has been replaced with that of the R5 (macrophage)-tropic Bal strain. Recombinant luciferase-expressing single-cycle pseudotyped HIV-1 particles were made upon co-transfection of 293T cells with pNL4-3*Luc*^+^*E*^- ^(obtained from the AIDS Repository Reagent Program) and pHCMV-G as described previously [89]. The latter molecular construct codes for the broad-host-range vesicular stomatitis virus envelope glycoprotein G (VSV-G) under the control of the human cytomegalovirus promoter [90]. Progeny viruses were also produced upon acute infection of purified CD4^+ ^T cells for 7 days with the R5-tropic clinical HIV-1 isolate 93TH054 (obtained from the AIDS Repository Reagent Program). The virus-containing supernatants were filtered through a 0.22 μm cellulose acetate syringe filter and normalized for virion content using a homemade p24 test. In this enzymatic assay, 183-H12-5C and 31-90-25 antibodies are used in combination to quantify p24 levels [91]. For experiments aimed at quantifying reverse transcription products and integrated viral DNA copies, NL4-3/Bal*env*-containing supernatants were filtered through a 0.22 μm cellulose acetate syringe filter, treated with DNase I (Roche) for 45 min at room temperature to prevent viral DNA carryover [92] and finally ultracentrifuged to eliminate enzyme in an Optima L-90K Beckman Coulter apparatus (Fullerton, CA) for 45 min at 28,000 rpm (100,000 × *g*) in a 70 Ti rotor.

### Virus transfer experiments

IM-MDDCs (5 × 10^4 ^in 100 μl of culture medium) were either left untreated or treated with one of the studied TLR agonists for 2 hours, washed twice and pulsed with virus preparations (2.5 ng of p24) for 1 hour at 37°C. In some studies, EFV (25 nM) was added to inhibit direct productive infection and left for 30 min before pulsing with viruses. Next, the virus-cell mixture was washed twice with phosphate-buffered saline (PBS) to remove free virions. For estimating early HIV-1 transfer, IM-MDDCs were co-cultured with autologous activated CD4^+ ^T lymphocytes at a 1:2 ratio in complete RPMI-1640 medium supplemented with rhIL-2 (30 U/ml) in 96-well flat-bottom tissue culture plates in a final volume of 200 μl. Cell-free supernatants (half of the medium) were harvested at day 2, 3 and 6 following initiation of the co-culture and kept frozen at -20°C. Virus production was estimated by measuring p24 levels in such culture supernatants.

### Virus infection studies

IM-MDDCs (5 × 10^4 ^in 100 μl of culture medium) were either left untreated or treated with a TLR agonist for 2 hours, washed twice and pulsed with virus preparations (2.5 ng of p24) for 1 hour at 37°C. In some studies, EFV (25 nM) was added to inhibit direct productive HIV-1 infection and left for 30 min before pulsing with viruses. Next, the virus-cell mixture was washed twice with PBS to remove free virions. Inverse kinetic was also performed where IM-MDDCs were first infected for 1 hour and then stimulated with a TLR2 agonist. Thereafter, IM-MDDCs were cultured in complete RPMI-1640 medium in 96-well flat-bottom tissue culture plates in a final volume of 200 μl. Supernatants (half of medium) were harvested either at day 3, 6 and 9 (infection with NL4-3/Bal*env*) or 5, 8 and 12 (infection with 93TH054) following HIV-1 infection and kept at -20°C until assayed for p24 contents. For studies aimed at defining the contribution of type-I IFNs, the same experimental procedure was followed except that the medium was supplemented with B18R (0.1 μg/ml). In some experiments, IM-MDDCs were infected with VSV-G pseudotyped reporter virus (2.5 ng of p24) for 2 hours and washed to remove free virions. Next, the virus-cell mixture was cultured for 48 hours before stimulation with the TLR2 agonist for 2 hours. Cells were then washed and luciferase activity was monitored 48 hours later.

### Quantification of IFNα/β

IM-MDDCs were either left untreated or treated for 6 hours with all studied TLR agonists. Next, levels of IFNα/β in cell-free supernatants were determined through the use of HEK-Blue™ IFNα/β cells according to the manufacturer's protocol (InvivoGen). These cells allow the detection of bioactive IFNα and IFNβ by monitoring the activation of the ISGF3 pathway. HEK-Blue™ IFNα/β cells are stably transfected with a SEAP promoter gene under the control of the IFNα/β-inducible ISG54 promoter. Stimulation of these cells with type-I IFNs activates the JAK/STAT/ISGF3 pathway and induces subsequently the secretion of SEAP in the supernatant. A standard curve of IFNα ranging from 1 to 250 Units/ml was used to quantify the amounts of type-I IFNs released in the culture medium.

### Electrophoresis and western blotting

IM-MDDCs (5 × 10^6^) were either left untreated or treated with a TLR agonist for 0, 2, 5, 15 and 30 min. For each time point, the equivalent of 2.5 × 10^4 ^cells was transferred into 2× sample buffer. Samples were boiled for 7 min and kept at -20°C until subjected to a western blot analysis. In brief, samples were loaded onto sodium dodecyl sulphate (SDS)-10% polyacrylamide gel electrophoresis (PAGE). Proteins were then transferred to Immobilon membranes (Millipore Corporation, Bedford, Massachussets, USA). Immunoblotting was performed first with anti-phospho-IκBα (dilution 1:1000) overnight at 4°C. Next, the membrane was stripped and blotted with anti-IκBα (dilution 1:1000) overnight at 4°C. To measure the amount of protein loaded in the gel, the membrane was stripped again and immunoblotted with anti-actin (dilution 1:5000) for 1 h at room temperature. Proteins were detected with an enhanced chemiluminescence reagent (Pierce) followed by exposure to Kodak films.

### Bio-Plex cytokine assay

A commercial Bio-Plex cytokine test that can detect and quantify 7 different cytokines (i.e. IL-1β, IL-6, TNFα, IL-12p70, MIP-1α, MIP-1β and RANTES) through the use of the Luminex^® ^100™ apparatus (Luminex Corporation, Austin, TX) was purchased from Bio-Rad Laboratories (Mississauga, ON). The Luminex technology is a bead array cytometric analyzer designed to study numerous analytes simultaneously by using spectrally distinct beads in a single well of a microtiter plate, using very small sample volumes (i.e. as little as 25 μl). Briefly, IM-MDDCs were either left untreated or treated for 8 and 24 hours at 37°C with the following stimuli: Pam3Csk4 (5 μg/ml), LPS (0.1 μg/ml), flagellin (5 μg/ml), R837 (5 μg/ml) and unmethylated DNA (5 μg/ml). Quantification was achieved by measuring concentrations of the studied cytokines and chemokines in cell-free supernatants according to the manufacturer's instructions.

### Virus entry assay

IM-MDDCs (4.5 × 10^6 ^in 1.8 ml of culture medium) were either left untreated or treated with the TLR2 or 4 agonist for 2 hours and washed twice. Then, cells (5 × 10^5 ^in 200 ul of complete culture medium) were pulsed with HIV-1 (12.5 ng of p24) for 15, 30 and 60 min at 37°C. The virus-cell mixture was washed extensively with PBS and treated with trypsin to remove uninternalized virions. Finally, cells were lysed with 250 μl of lysis buffer (HEPES 20 mM, NaCl 150 mM and Triton 0.5%). Viral entry was estimated by measuring p24 levels in lysed cells.

### Quantification of reverse transcription products and integrated viral DNA copies

IM-MDDCs (1 × 10^6^) were either left untreated or treated with the TLR2 or TLR4 agonist for 2 hours and pulsed with DNaseI treated NL4-3/Bal*env *(100 ng of p24 per 1 × 10^6 ^cells) for 1 hour and washed twice. IM-MDDCs were cultured for 6, 24 and 48 hours. Then, genomic DNA was extracted using the DNeasy Blood & Tissue Kit (QIAGEN, Mississauga, ON). Integrated proviral DNA copies were quantified using a combined *Alu*-HIV-1 PCR and real-time PCR assay as described by Suzuki and colleagues [93]. Briefly, genomic DNA (100 ng) was first amplified with an *Alu*-sequence-specific sense primer and HIV-1-specific antisense primer (i.e. M661) [94]. Next, 5 μl of 25-fold diluted PCR products were subjected to a real-time PCR assay in 25 μl reaction containing 2× TaqMan Universal PCR Master Mix (Applied Biosystems, Foster City, CA), 2 μM of the sense primer M667, 2 μM of the antisense primer AA55, and 0.3 μM of the TaqMan probe HIV-5'-carboxyfluorescein (Biosearch Technologies, Novato, USA). The cycling conditions included a hot start (50°C for 2 min and 95°C for 10 min), followed by 40 cycles of denaturation (95°C for 1 min) and extension (63°C for 1 min) with end point acquisition. NL4-3/Bal*env *DNA was used for the standard curve (i.e., from 469 to 30,000 copies). HIV-1 standards contain 1 ng of DNA from uninfected cells as carrier. For quantification of early and late reverse transcripts, 25 ng of DNA were subjected to a real-time PCR assay in 25 μL reaction containing 2× TaqMan Universal PCR Master Mix (Applied Biosystems, Foster City, CA), 1 μM of the sense primer M667, 1 μM of the antisense primer M661 (late RT) or AA55 (total RT), and 0.3 μM of the TaqMan probe HIV-5'-carboxyfluorescein (Biosearch Technologies, Novato, USA) [39]. NL4-3Bal*env *DNA was used for the standard curve (i.e. from 235 to 15,000 copies). HIV-1 standards contained 25 ng of DNA from uninfected cells as carrier. The amounts of early reverse transcripts were obtained by subtracting the late reverse transcripts from the total reverse transcripts.

### Statistical analysis

Statistical analyses were carried out according to the methods outlined in Zar and Sokal and Rohlf. Briefly, homoscedasticity was determined using the variance ratio test and the means were compared using a single factor ANOVA followed by appropriate *post hoc *multiple comparisons (Tukey's or Dunnett's). P values lower than 0.05 were considered highly significant. For the percentages of inhibition, statistical analysis was performed by using arcsin transformation followed by a Student's *t*-test. Results from three or more experiments were always used for these analyses. Computations were carried out using GraphPad PRISM ^® ^version 3.03 statistical software.

## Competing interests

The authors declare that they have no competing interests.

## Authors' contributions

ST performed the experiments and prepared Figures 1 to 8. RF performed the PCR experiments and prepared Figure 7. ST and MRT analyzed the data and wrote the manuscript. MJT supervised and coordinated the study and finalized the manuscript. All authors read and approved the final manuscript.
